# Supplementary material for: Investigating wave solutions and impact of nonlinearity: Comprehensive study of the KP-BBM model with bifurcation analysis
Source: PLoS One. 2024 May 2;19(5):e0300435. doi: 10.1371/journal.pone.0300435 (PMC11065286; doi:10.1371/journal.pone.0300435)
Supplement: S1 File — (DOCX) [file pone.0300435.s001.docx]

**Investigating wave solutions and impact of nonlinearity: Comprehensive study of the KP-BBM model with bifurcation analysis**

S M Rayhanul Islam^1^, Kamruzzaman Khan^1, 2^

^1^Department of Mathematics, Pabna University of Science and Technology, Pabna-6600, Bangladesh.

^2^School of Science and Technology, University of New England, Armidale, NSW 2351, Australia.

**S2 File. Unified method.**

Consider the NLEEs in the generic form:

$\mathfrak{I(}u,u_{t},u_{x},u_{tt},u_{xx},u_{xt},\ldots)=0$, (A1)

where $u(x,y,t)$ is an unknown function, $\mathfrak{I}$ is a polynomial in $u=u(x,y,t)$. Through the wave transformation

$u\left( x,y,t \right)= \varphi(\xi)$,$\xi=\lambda x+\mu y-\sigma t$, (A2)

In Eq. (A2.), the coefficients $\lambda$ and $\mu$ represents the width of the soliton in $x$ and $y$-directions and $\sigma$ is the speed of soliton. Eq. (A1) is transformed into the subsequent nonlinear equation as

$\mathfrak{I(}\varphi,\varphi^{'},\varphi^{''},\cdots)=0$, (A3)

In line with unified approach, the solitary wave solution of (A3) is estimated as

$\varphi\left( \xi\right)=A_{0}+\sum_{j=1}^{N} {[A}_{j}w^{j}+B_{j}w^{-j}]$, (A4)

where $A_{j},B_{j}(j=1, 2, 3...N)$ are contants and $w=w( \xi)$ satisfies the following equation:

$w^{'}\left( \xi\right)=w^{2}\left( \xi\right)+k$, (A5)

where $w^{'}=\frac{dw}{d\xi}$ and $k$ is a parameter. The following solutions of Eq. (A5) are established in accordance with the values of $k$:

**Group 1**: For $k<0$, the hyperbolic solutions of Eq. (A5) are:

$w\left( \xi\right)=\frac{\sqrt{-\left( ϰ^{2}+\mathcal{l}^{2} \right)k}-ϰ\sqrt{-k}\cosh\left( 2\sqrt{-k} \left( \xi\mathcal{+H} \right) \right)}{ϰsinh \left( 2\sqrt{-k} \left( \xi\mathcal{+H} \right) \right)+\mathcal{l}}$,

$w\left( \xi\right)=\frac{-\sqrt{-\left( ϰ^{2}+\mathcal{l}^{2} \right)k}-ϰ\sqrt{-k}\cosh\left( 2\sqrt{-k}\left( \xi\mathcal{+H} \right) \right)}{ϰ \sinh\left( 2\sqrt{-k} \left( \xi\mathcal{+H} \right) \right)+\mathcal{l}}$,

$w\left( \xi\right)=\sqrt{-k}+\frac{-2ϰ\sqrt{-k}}{ϰ+\cosh\left( 2\sqrt{-k} \left( \xi\mathcal{+H} \right) \right)-\sinh\left( 2\sqrt{-k} \left( \xi\mathcal{+H} \right) \right)}$,

$w\left( \xi\right)=-\sqrt{-k}+\frac{2ϰ\sqrt{-k}}{ϰ+\cosh\left( 2\sqrt{-k}\left( \xi\mathcal{+H} \right) \right)+\sinh\left( 2\sqrt{-k}\left( \xi\mathcal{+H} \right) \right)}$.

**Group 2**: For $k>0$, the trigonometric solutions of Eq. (A5) are:

$w\left( \xi\right)=\frac{\sqrt{\left( ϰ^{2}-\mathcal{l}^{2} \right)k}-ϰ\sqrt{k}\cos\left( 2\sqrt{k}\left( \xi\mathcal{+H} \right) \right)}{ϰ \sin\left( 2\sqrt{k}\left( \xi\mathcal{+H} \right) \right)+\mathcal{l}}$,

$w\left( \xi\right)=\frac{-\sqrt{\left( ϰ^{2}-\mathcal{l}^{2} \right)k}-ϰ\sqrt{k}\cos\left( 2\sqrt{k}\left( \xi\mathcal{+H} \right) \right)}{ϰ \sin\left( 2\sqrt{k}\left( \xi\mathcal{+H} \right) \right)+\mathcal{l}}$,

$w\left( \xi\right)=i\sqrt{k}+\frac{-2iϰ\sqrt{k}}{ϰ+\cos\left( 2\sqrt{k} \left( \xi\mathcal{+H} \right) \right)-i\sin\left( 2\sqrt{k}\left( \xi\mathcal{+H} \right) \right)}$,

$w\left( \xi\right)=-i\sqrt{k}+\frac{2iϰ\sqrt{k}}{ϰ+\cos\left( 2\sqrt{k}\left( \xi\mathcal{+H} \right) \right)+i\sin\left( 2\sqrt{l}\left( \xi\mathcal{+H} \right) \right)}$.

**Group 3**: For $k=0$, the solutions of Eq. (A5) are:

$w\left( \xi\right)=-\frac{1}{\xi\mathcal{+H}}$ .

Here, the arbitrary constants $ϰ$ and $\mathcal{l}$ are real, and $\mathcal{H}$ is any constant. Introducing solution (A4) and (A5) in Eq. (A3) and summing up all the coefficients of $w^{j}=(-N\leq j\leq N)$ to zero yield a set of algebraic equations for $A_{j}$, $B_{j}$, $\sigma$ and $k$. Locating $A_{j}$, $B_{j}$, $\sigma$ and $k$ into (A4) and using the solutions of (A5), it can be obtained scores of soliton solutions of Eq. (A1) directly based on the value of$k$ [3, 28].
