# Supplementary material for: Investigating wave solutions and impact of nonlinearity: Comprehensive study of the KP-BBM model with bifurcation analysis
Source: PLoS One. 2024 May 2;19(5):e0300435. doi: 10.1371/journal.pone.0300435 (PMC11065286; doi:10.1371/journal.pone.0300435)
Supplement: S2 File — (DOCX) [file pone.0300435.s002.docx]

**Investigating wave solutions and impact of nonlinearity: Comprehensive study of the KP-BBM model with bifurcation analysis**

S M Rayhanul Islam^1^, Kamruzzaman Khan^1, 2^

^1^Department of Mathematics, Pabna University of Science and Technology, Pabna-6600, Bangladesh.

^2^School of Science and Technology, University of New England, Armidale, NSW 2351, Australia.

**S1 File. Advanced auxiliary equation method.**

Consider a NLEEs in the following structure,

$Ɲ(u,u_{t},u_{x},u_{xx},u_{tx},u_{tt},\ldots\ldots)=0,$ (B1)

where $Ɲ$ is a nonlinear polynomial function of wave function $u(x,t)$, and including its disparate partial derivatives. We suppose that

$u\left( x,y,t \right)= \varphi(\xi)$,$\xi=\lambda x+\mu y-\sigma t$. (B2)

In Eq. (B2), the coefficients $\lambda$ and $\mu$ represents the width of the soliton in $x$ and $y$-directions and $\sigma$ is the speed of soliton. Eq. (B2) converts to Eq. (B1) into a nonlinear ordinary differential equation as

$£( \varphi,\varphi^{'},\varphi^{''},\cdots\cdots\cdots)=0,$ (B3)

among them, prime represents the derivative of $\xi$. According to the AAE method, the solution of Eq. (B3) is conjecture to be

$\varphi\left( \xi\right)=\sum_{i=0}^{N} c_{i}a^{ig(\xi)},$ (B4)

where the constants $c_{0},c_{1},c_{2},\ldots\ldots,c_{N}$ are calculated, such that $c_{N}\neq0$, according to the balanced theorem, we get value $N$ in Eq. (B4) and $g(\xi)$ are the solution of the equation as

$g^{'}\left( \xi\right)=\frac{1}{\ln\left( a \right)}\left\{ \alpha a^{-g\left( \xi\right)}+\beta+\gamma a^{g\left( \xi\right)} \right\}.$ (B5)

In this step, we substitute the Eq. (B4) and Eq. (B5) into Eq. (B3) and we get an algebraic equation which are equated left and right side based on powers of $a^{ig(\xi)},(i=0,1,2,3\ldots\ldots)$. As a result, we gain an algebraic equation, solving these equations and we find out the values of $c_{0,}c_{1},c_{2,}\ldots\ldots c_{N}$ and $\sigma$. The solutions of Eq. (B5) are obtained as follows:

**Case-1:** when $\beta^{2}-4\alpha\gamma<0$ and $\gamma\neq0,$

$a^{g(\xi)}=\frac{-\beta}{2\gamma}+\frac{\sqrt{4\alpha\gamma-\beta^{2}}}{2\gamma}\tan\left( \frac{\sqrt{4\alpha\gamma-\beta^{2}}}{2} \xi\right)$ (2.5.1)

and

$a^{g(\xi)}=\frac{-\beta}{2\gamma}-\frac{\sqrt{4\alpha\gamma-\beta^{2}}}{2\gamma}\cot\left( \frac{\sqrt{4\alpha\gamma-\beta^{2}}}{2} \xi\right)$.

**Case-2:** when $\beta^{2}-4\alpha\gamma>0$ and $\gamma\neq0,$

$a^{g(\xi)}=\frac{-\beta}{2\gamma}-\frac{\sqrt{4\alpha\gamma-\beta^{2}}}{2\gamma}\tanh\left( \frac{\sqrt{4\alpha\gamma-\beta^{2}}}{2} \xi\right)$

and

$a^{g(\xi)}=\frac{-\beta}{2\gamma}-\frac{\sqrt{4\alpha\gamma-\beta^{2}}}{2\gamma}\coth\left( \frac{\sqrt{4\alpha\gamma-\beta^{2}}}{2} \xi\right)$.

**Case-3:** when $\beta^{2}+4\alpha^{2}<0,\gamma\neq0$ and $\gamma=-\alpha,$

$a^{g(\xi)}=\frac{\beta}{2\alpha}-\frac{\sqrt{-\beta^{2}-4\alpha^{2}}}{2\alpha}\tan\left( \frac{\sqrt{{-\beta}^{2}-4\alpha^{2}}}{2}\xi\right)$

and

$a^{g(\xi)}=\frac{\beta}{2\alpha}+\frac{\sqrt{-\beta^{2}-4\alpha^{2}}}{2\alpha}\cot\left( \frac{\sqrt{{-\beta}^{2}-4\alpha^{2}}}{2}\xi\right)$.

**Case-4:** when $\beta^{2}+4\alpha^{2}>0,\gamma\neq0$ and $\gamma=-\alpha,$

$a^{g(\xi)}=\frac{\beta}{2\alpha}+\frac{\sqrt{\beta^{2}+4\alpha^{2}}}{2\alpha}\tanh\left( \frac{\sqrt{\beta^{2}+4\alpha^{2}}}{2}\xi\right)$

and

$a^{g(\xi)}=\frac{\beta}{2\alpha}+\frac{\sqrt{\beta^{2}+4\alpha^{2}}}{2\alpha}\coth\left( \frac{\sqrt{\beta^{2}+4\alpha^{2}}}{2}\xi\right)$.

**Case-5:** when $\beta^{2}-4\alpha^{2}<0$ and $\gamma=\alpha,$

$a^{g(\xi)}=\frac{-\beta}{2\alpha}+\frac{\sqrt{{-\beta}^{2}+4\alpha^{2}}}{2\alpha}\tan\left( \frac{\sqrt{{-\beta}^{2}+4\alpha^{2}}}{2} \xi\right)$

and

$a^{g(\xi)}=\frac{-\beta}{2\alpha}-\frac{\sqrt{{-\beta}^{2}+4\alpha^{2}}}{2\alpha}\cot\left( \frac{\sqrt{{-\beta}^{2}+4\alpha^{2}}}{2} \xi\right)$.

**Case-6:** when $\beta^{2}-4\alpha^{2}>0$ and $\gamma=\alpha,$

$a^{g(\xi)}=\frac{-\beta}{2\alpha}-\frac{\sqrt{\beta^{2}-4\alpha^{2}}}{2\alpha}\tanh\left( \frac{\sqrt{\beta^{2}-4\alpha^{2}}}{2} \xi\right)$

and

$a^{g(\xi)}=\frac{-\beta}{2\alpha}-\frac{\sqrt{\beta^{2}-4\alpha^{2}}}{2\alpha}\coth\left( \frac{\sqrt{\beta^{2}-4\alpha^{2}}}{2} \xi\right)$.

**Case-7:** when $\beta^{2}=4\alpha\gamma,$

$a^{g(\xi)}=-\frac{2+\beta\xi}{2\gamma\xi}$.

**Case-8:** when $\alpha\gamma<0,\beta=0$ and $\gamma\neq0,$

$a^{g(\xi)}=-\sqrt{\frac{-\alpha}{\gamma}}\tanh\left( \sqrt{-\alpha\gamma}\xi\right)$

and

$a^{g(\xi)}=-\sqrt{\frac{-\alpha}{\gamma}}\coth\left( \sqrt{-\alpha\gamma}\xi\right)$.

**Case-9:** when $\beta=0$ and $\alpha=-\gamma,$

$a^{g(\xi)}=\frac{1+e^{(-2\gamma\xi)}}{-1+e^{(-2\gamma\xi)}}$.

**Case-10:** when $\alpha=\gamma=0,$

$a^{g(\xi)}=\cosh\left( \beta\xi\right)+\sinh\left( \beta\xi\right)$.

**Case-11:** when $\beta=\alpha=K$ and $\gamma=0,$

$a^{g(\xi)}=e^{K\xi}-1$.

**Case-12:** when $\beta=\gamma=K$ and $\alpha=0,$

$a^{g(\xi)}=\frac{e^{K\xi}}{1-e^{K\xi}}$.

**Case-13:** when $\beta=(\alpha+\gamma),$

$a^{g(\xi)}=-\frac{1-\alpha e^{(\alpha-\gamma)\xi}}{1-{\gamma e}^{(\alpha-\gamma)\xi}}$.

**Case-14:** when $\beta=-(\alpha+\gamma),$

$a^{g(\xi)}=\frac{\alpha-e^{(\alpha-\gamma)\xi}}{\gamma-e^{(\alpha-\gamma)\xi}}$.

**Case-15:** when $\alpha=0,$

$a^{g(\xi)}=\frac{\beta e^{\beta\xi}}{1-\gamma e^{\beta\xi}}$.

**Case-16:** when $\gamma=\beta=\alpha\neq0,$

$a^{g(\xi)}=\frac{1}{2}\{\sqrt{3} tan\left( \frac{\sqrt{3}}{2}\alpha\xi\right)-1\}$.

**Case-17:** when $\gamma=\beta=0,$

$a^{g(\xi)}=\alpha\xi$.

**Case-18:** when $\alpha=\beta=0,$

$a^{g(\xi)}=\frac{-1}{\gamma\xi}$.

**Case-19:** when $\gamma=\alpha$ and $\beta=0,$

$a^{g(\xi)}=\tan\left( \alpha\xi\right)$.

**Case-20:** when $\gamma=0,$

$a^{g(\xi)}=e^{\betaΩ}-\frac{a}{b}$.

Relieving these values of $c_{i}\left( i=0,1,2,\ldots.,N \right),\alpha,\beta,\gamma$ and function$g(\xi)$ into Eq. (B4), produce numerous soliton solutions to the Eq. (B1) [6, 29].
