# Supplementary material for: Investigating wave solutions and impact of nonlinearity: Comprehensive study of the KP-BBM model with bifurcation analysis
Source: PLoS One. 2024 May 2;19(5):e0300435. doi: 10.1371/journal.pone.0300435 (PMC11065286; doi:10.1371/journal.pone.0300435)
Supplement: Attachment — Submitted filename: Comments.pdf [file pone.0300435.s003.pdf]

Review Report

TITLE: Investigating wave solutions and impact of nonlinearity: Comprehensive study of the KP-BBM model with bifurcation analysis.

In this article, authors have explored the (2+1)-dimensional Kadomtsev-Petviashvili-Benjamin-Bona Mahony equation using two effective methods via unified and advanced auxiliary equation schemes to derive precise wave solutions. The obtained numerical results are reliable, straightforward, and potent tools for analyzing various nonlinear evolution equations. The article is interesting and is on average level. However, I suggest some necessary corrections as follows:

1- What are the applications of this work?

2- Add statement of novelty.

3- P-1 (+14): replace “figures” with “Figures”.

4- Figures (6-9) are not cited.

5- Improve the resolution of all Figures.

6- On line 382, there should be space after “equilibrium point”.

I recommend minor revision for this article.
